# Association between different sensory modalities based on concurrent time series data obtained by a collaborative reservoir computing model

**DOI:** 10.1038/s41598-023-27385-x

**Published:** 2023-01-04

**Authors:** Itsuki Kanemura, Katsunori Kitano

**Affiliations:** 1 Graduate School of Information Science and Engineering, Ritsumeikan University, 1-1-1, Nojihigashi, Kusatsu, Shiga 5258577 Japan; 2 Department of Information Science and Engineering, Ritsumeikan University, 1-1-1, Nojihigashi, Kusatsu, Shiga 5258577 Japan

**Keywords:** Computational neuroscience, Sensory processing

## Abstract

Humans perceive the external world by integrating information from different modalities, obtained through the sensory organs. However, the aforementioned mechanism is still unclear and has been a subject of widespread interest in the fields of psychology and brain science. A model using two reservoir computing systems, i.e., a type of recurrent neural network trained to mimic each other's output, can detect stimulus patterns that repeatedly appear in a time series signal. We applied this model for identifying specific patterns that co-occur between information from different modalities. The model was self-organized by specific fluctuation patterns that co-occurred between different modalities, and could detect each fluctuation pattern. Additionally, similarly to the case where perception is influenced by synchronous/asynchronous presentation of multimodal stimuli, the model failed to work correctly for signals that did not co-occur with corresponding fluctuation patterns. Recent experimental studies have suggested that direct interaction between different sensory systems is important for multisensory integration, in addition to top-down control from higher brain regions such as the association cortex. Because several patterns of interaction between sensory modules can be incorporated into the employed model, we were able to compare the performance between them; the original version of the employed model incorporated such an interaction as the teaching signals for learning. The performance of the original and alternative models was evaluated, and the original model was found to perform the best. Thus, we demonstrated that feedback of the outputs of appropriately learned sensory modules performed the best when compared to the other examined patterns of interaction. The proposed model incorporated information encoded by the dynamic state of the neural population and the interactions between different sensory modules, both of which were based on recent experimental observations; this allowed us to study the influence of the temporal relationship and frequency of occurrence of multisensory signals on sensory integration, as well as the nature of interaction between different sensory signals.

## Introduction

The brain uses numerous sensory inputs to recognize the external world and acquire knowledge. In addition, handling multiple pieces of information in an integrated manner increases the reliability of perceiving the external world and knowledge acquisition. Researchers have investigated this phenomenon, termed sensory integration or multisensory integration, in the fields of psychology, medicine, neuroscience, and artificial intelligence.

In psychology, the "McGurk effect" describes the perception of a third phoneme when people listen to one phoneme being vocalized while simultaneously seeing another phoneme being vocalized in a video^1^. The co-occurrence and combination of specific visual and auditory stimuli exert a significant impact on perception^2,3^. In neuroscience, most experimental studies employ the experimental design requiring perceptual decision-making to distinguish between multimodal and unimodal sensory effects^4,5^. The models based on Bayesian decision theory succeed in explaining the mechanism underlying multisensory integration, especially the mechanism of perceptual enhancement for combinations of multiple sensory stimuli. The models based on the drift–diffusion theory could explain reaction time in perceptual decision-making of multisensory stimuli^6,7^. These models are based on the hierarchical structure, namely feedforward structure, in which sensory information is processed in the corresponding unimodal sensory system (e.g., visual cortex) and then transferred to association areas (via a single pathway) for integration with other types of sensory information. According to evidence that the view of the feedforward system is not necessarily consistent with the results of perturbation studies, in which a relevant brain region is inactivated, systems for multimodal sensory integration consist of multiple pathways and are more multifaceted than the feedforward view^8^. Such interaction between sensory modalities and integration of sensory information through these pathways before it reaches the association cortex would allow for faster perception than the conventional feedforward processing. In addition, because responses of single neurons during multisensory integration are heterogenous, it is also suggested that the contribution of population-level dynamics to the mechanism should be considered^8^. From the perspective of computer science, one made attempts to model a visual system based on the convolutional neural network (CNN)^9^ and the mechanism for sound source localization^10^. Regarding audio-visual integration, the model with self-supervised learning, CNN, and Bayesian theory proposed the mechanism that utilizes correspondence and temporal coincidence between features, each of which is extracted from visual or auditory stimuli^11–24^.

Sensory information comprises an uninterrupted time series, and sensory organs transmit it as a continuous signal to downstream areas. Sensory neurons during such a sensory stimulus presentation exhibit dynamic firing patterns, but not steady firing intensities^25^. In the early stage of multimodal integration, sensory organs or low-level brain regions, such as the retina/primary visual cortex for the visual system or cochlea/primary auditory cortex for the auditory system, need to extract useful information from such a continuous signal and to encode extracted information as a dynamical state of neural activity. Most of the studies concerning multisensory integration have focused on how to utilize acquired information, i.e., the features obtained after processing the continuous signals, but not on how to extract information from continuous signals^26,27^. In addition, the encoded sensory information is assumed to be represented by steady firing rates of a neural population (i.e., a static vector), not by a dynamical state of them. Indeed, the time-varying activity of a neural population has been observed in various brain regions and showed its relevance to brain functions^28^. Perception of multisensory stimuli depends on the timings of stimuli such as stimuli presented with a certain delay^3^. This dependence of multisensory integration on the timings could be achieved only with the model based on the dynamical state. Furthermore, because the process is carried out in an unsupervised manner, it is likely that frequent simultaneous presentation of signals from different sensory modes should serve as the cue. Although how multimodal information is constructed from continuous signals has been studied^22^ from the perspective of machine learning, most of the studies utilize a steady state of the neural population, rather than a dynamical state, as the encoding relies on an artificially layered structure that is not seen in the brain^11–24^.

In this study, we aimed to propose a potent model for understanding the mechanism of multisensory integration in the early stage implemented by the brain based on the previously proposed model for chunking^29^. The model is based on reservoir computing (RC) because RC is a recurrent network model that handles time series data^30,31^. While most models for chunking focus on the transition probability between elements that make up a chunk^32–35^, the model utilizes trajectories of states of the neural network responding to sequential inputs. Because sensory signals are not always comprised of separable elements such as letters in a string, the model is more likely to provide a plausible mechanism. Furthermore, because the neuronal network in sensory organs, such as the retina for the visual system, rarely shows experience-dependent plasticity, RC in which recurrent connections are fixed is structurally closer to the system engaging in sensory processing than other types of recurrent neural networks in which all connections are supposed to be modified for learning. Similarly, a reservoir computing model based on predictive coding (PCRC) was proposed^36^. The PCRC model was constructed such that the sensory information of each modality was supposed to be encoded as predicted information, namely, the encoding neural activity is supposed to reproduce the input. Therefore, in the model, each sensory input is essentially unaffected by the other types of sensory signals, and interaction between different sensory modalities was not considered. On the other hand, the proposed model enables us to consider the effect of direct interactions between sensory modules, which has been suggested by several studies^37–39^. In this study, we intended to apply the previously proposed model^29^ to realize information integration between different modalities through the segmentation of specific stimuli co-occurrence in repeatedly presented time series data and to encode it as a dynamical state.

## Results

### Collaborative multimodal RC model

**Figure 1:**
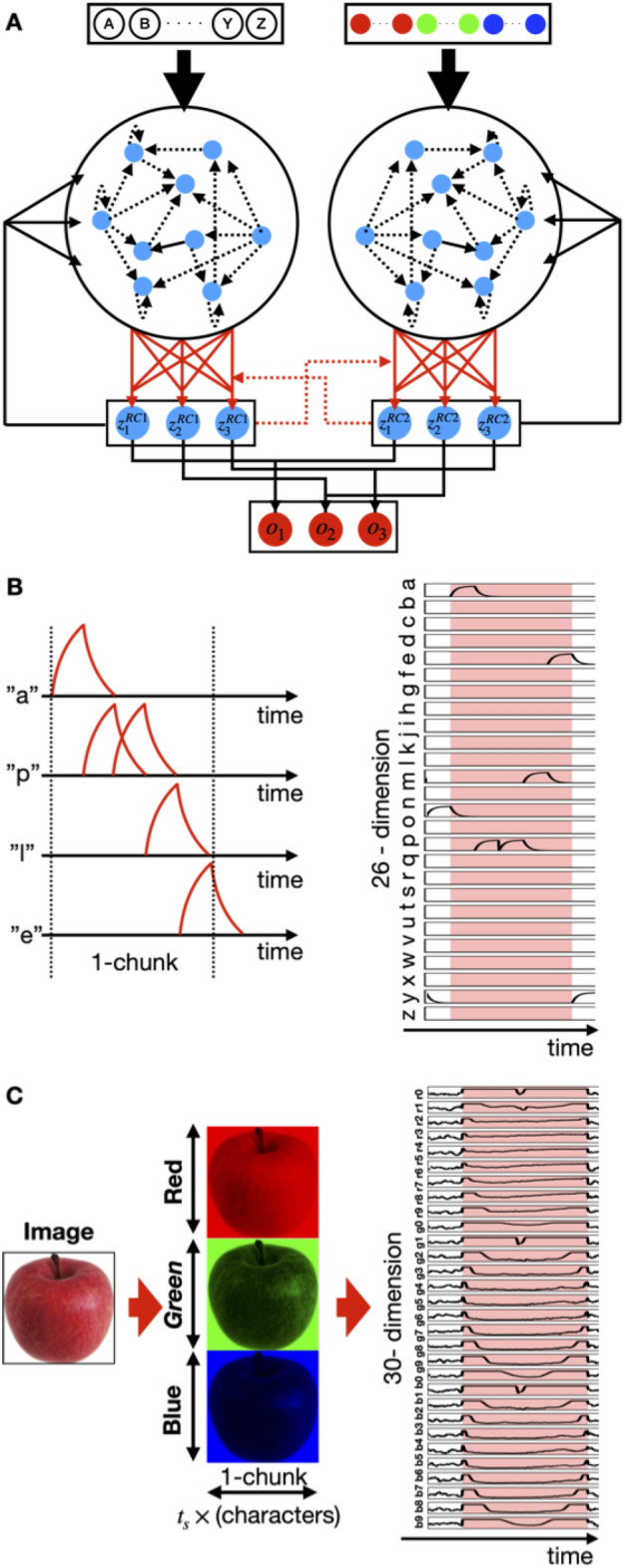
Collaborative multimodal reservoir computing model and signals used. (**A**) In the collaborative multimodal reservoir computing (RC) model, two non-identical reservoirs are activated by different input neurons' activity. The readout weights of each RC system undergo supervised learning with a teaching signal generated by the output of the partner network. Each input neuron of RC receives different modality inputs. In this simulation, we used a text-tone signal and an image signal. (**B**) Example responses are provided for input neurons to the text-tone modality signal. (**C**) Example responses are provided for input neurons to the image modality signal. Our demonstration uses three chunks, with each chunk displaying a fruit, namely an apple, grape, and banana.

We demonstrated the performance of the proposed collaborative RC model for multimodal time-series data^29^. In the brain, there are different systems, each of which is responsible for the processing of a specific sensory signal, such as signals from the visual system or the auditory system. Therefore, in the model used in this study, two RC networks, each of which is later referred to as an RC module, interacted with each other by feeding their output to the other module for learning. As described below, the model received different modal inputs (images and text tones) to be integrated, each of which is processed by an RC module that corresponds to a sensory system. Each RC module consisted of rate-based neurons in a recurrent network and linear readout units in the output layer. An RC module received the time-varying inputs of a modality, which differed among the modules (Fig. 1A). A neuron in a reservoir received input from one of the input neurons. All neurons were mutually connected within each reservoir, and a portion of these neurons projected to the readout units, which projected back to all neurons belonging to similar RC modules. The two reservoirs had different recurrent wiring patterns and were not identical. Readout activity z(t) was determined by the weighted sum of the activities ${\varvec{r}}\left(t\right)$ of the reservoir neurons projecting to the readout:

1 $${\varvec{z}}\left({\varvec{t}}\right)={{\varvec{W}}}^{{\varvec{o}}{\varvec{u}}{\varvec{t}}}{\varvec{r}}\left({\varvec{t}}\right).$$

In addition, we set a higher layer in which the units received outputs of the readout units in the RC modules. ${o}_{i}\left(t\right)$ denotes the activity of a unit in the association layer, used to calculate the accuracy rate (refer to “Methods”). The weight vector ${{\varvec{W}}}^{{\varvec{o}}{\varvec{u}}{\varvec{t}}}$ was modified through the first-order reduced and controlled error (FORCE) learning algorithm^40^, whereas recurrent and feedback connections were non-plastic because the model could solve the present task without modifying these connections. In our model, each RC used the teaching signal generated by the output ${\varvec{z}}\left(t\right)$ of the partner network through the learning process of the FORCE algorithm, which enabled the model to demonstrate unsupervised learning^29^.

During the demonstration, we used chunks that consisted of modal input pairs, which mimicked the multimodal sensory inputs. In the present study, one RC received an image, whereas the other received text comprising a short tone signal representing a character (Fig. 1B). Text inputs were implemented as the input with a time course different from that of images. A letter input transiently activates the corresponding input neuron to the letter, that is, the number of input neurons equals the number of letters. Random sequence inputs were generated by randomly chosen letters, the length of which ranges from 5 to 8. Contrarily, after scaling up or down to an identical size, the image was decomposed into RGB components (Fig. 1C). Subsequently, each of these components was divided into 10 sections from top to bottom. A section was scanned from left to right in a window, followed by pooling the scanned image within the window. Eventually, an image was converted into a 30-dimensional series of data and the length of the scanned image was converted into time series data, according to the length of the text tone inputs. Thus, the number of input neurons coincided with 30 neurons. We used a Gaussian noise image as the random sequence, which was processed similar to image signals. Image signals can be constructed in other ways; for example, an image is encoded as a static vector, following which the value of an element in the vector is fed as a static input throughout the duration. However, our goal was to determine if the proposed model achieved responses to simultaneously presented time-varying signals with varying characteristics because they originated from different modalities. Therefore, we composed input signals such that a set of co-current time series represented a chunk, each of which originated from different modalities. We intended to demonstrate the behavior of the proposed model in response to inputs without co-occurrence.

### Learning of multimodal multiple chunks

**Figure 2:**
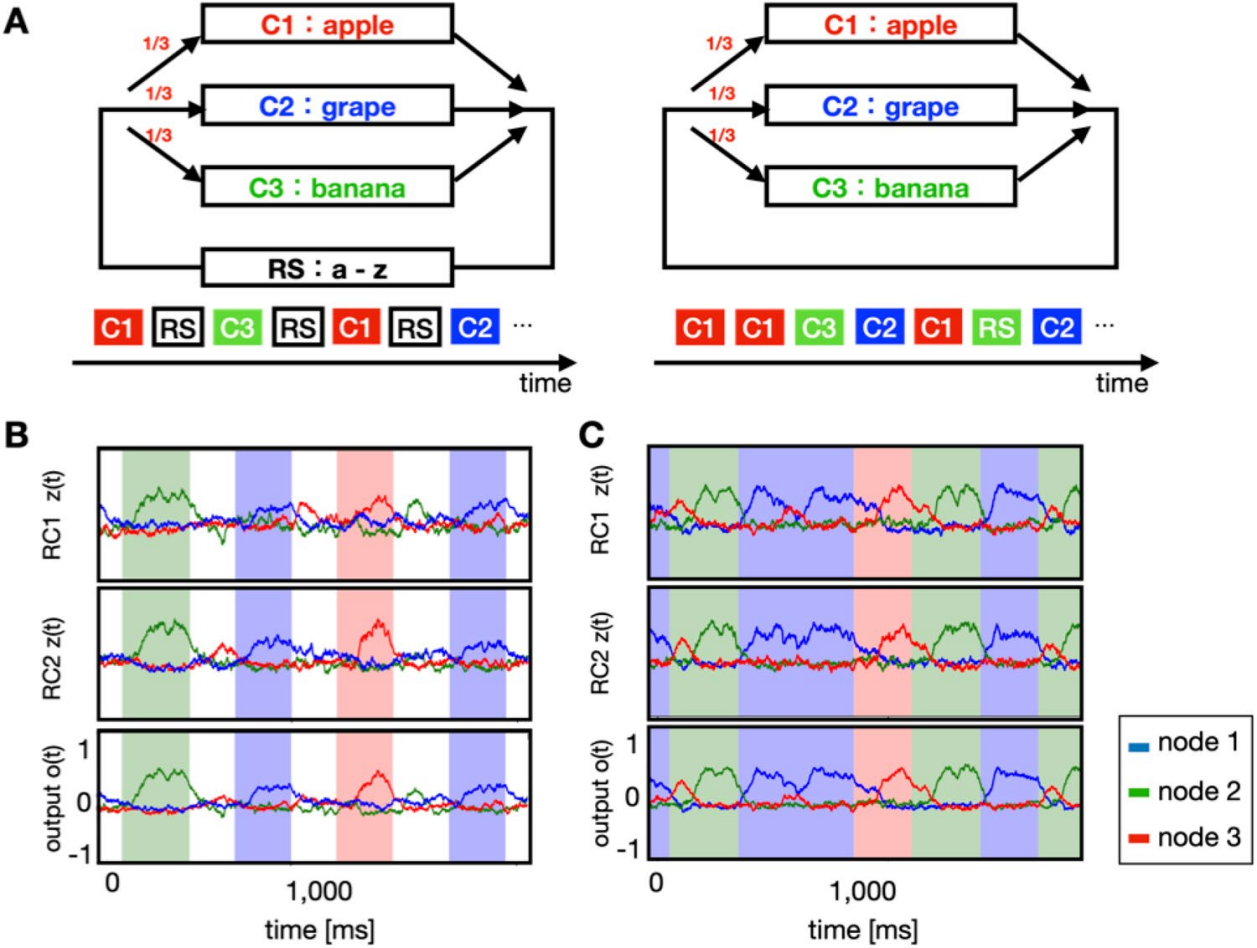
Learning of multimodal multiple chunks. (**A**) Left: Three chunks of apple (red), grape (blue), and banana (green) separated by random sequences recur at equal frequencies in the input. Right: These chunks are repeated without random sequence intervals. (**B**) The activity of the output units upon presenting each chunk while using input signals separated by random sequence intervals. The red, green, and blue shades indicate periods of learned input “apple,” input “banana,” and input “grape,” respectively. (**C**) The behavior of the output units upon presenting each chunk with input signals not separated by intervals. The red, green, and blue shades indicate periods of learned input “apple,” input “banana,” and input “grape,” respectively.

We demonstrated the mechanism by which the model learned multiple chunks. The input was made by alternating input sequence and a chunk that was randomly chosen with the same probability among the three. (Fig. 2A, left). In the model, the number of the readout units was set to three, ${z}_{i}^{RCj}$ for the *j*th RC module (*i* = 1, 2, 3). The teaching signals are defined by the following equations.

2 $${f}_{i}^{{\text{RC}}1}\left(t\right)={\left[\mathrm{tanh}\left(\frac{{\widehat{z}}_{i}^{RC2}\left(t\right)-\upgamma \left({\sum }_{j}{\widehat{z}}_{j}^{RC2}-{\widehat{z}}_{i}^{RC2}\right)}{\upbeta }\right)\right]}_{+},\left(i,j=\mathrm{1,2},3\right),$$

3 $${f}_{i}^{{\text{RC}}2}\left(t\right)={\left[\mathrm{tanh}\left(\frac{{\widehat{z}}_{i}^{RC1}\left(t\right)-\upgamma \left({\sum }_{j}{\widehat{z}}_{j}^{RC1}-{\widehat{z}}_{i}^{RC1}\right)}{\upbeta }\right)\right]}_{+},\left(i,j=\mathrm{1,2},3\right).$$

We trained the model by using input sequences in which three chunks appeared randomly and consecutively with equal probability (Fig. 2A, right). Thus, a similar RC system could easily learn multiple chunks (Fig. 2C). The selective response to each chunk was self-organized by stimulus co-occurrence upon each RC receiving input from different modalities.

Next, we investigated the mechanism by which activities of reservoir neurons encoded chunks. We trained the network on sequences containing three chunks and random sequences (Fig. 2A left). Each output neuron responded to the presentation of a specific chunk (Fig. 2B). Concerning reservoir neurons, some reservoir neurons responded to more than one chunk in each reservoir. As learning proceeds, the readout units could be classified into three groups, each of which exhibits selectivity to a specific chunk. The readout units showed time-locked responses to the chunk presentation (Fig. 2B). The activity emerged from the onset of the chunk presentation and then fell to the end of the presentation. This delayed response is presumably due to the teacher signal that is increased as selectivity becomes stronger. Readout activities of RC1 and RC2 in response to chunk presentation were considerably similar to each other. The detailed connection patterns within the reservoir units differed between the modules. Furthermore, these modules received time-varying inputs with different characteristics owing to dissimilar modalities. Nonetheless, the teacher signal based on the readout activity was provided to the partner RC module, such that the readout activity tended to imitate that of the partner RC module, thereby generating similar outputs.

**Figure 3:**
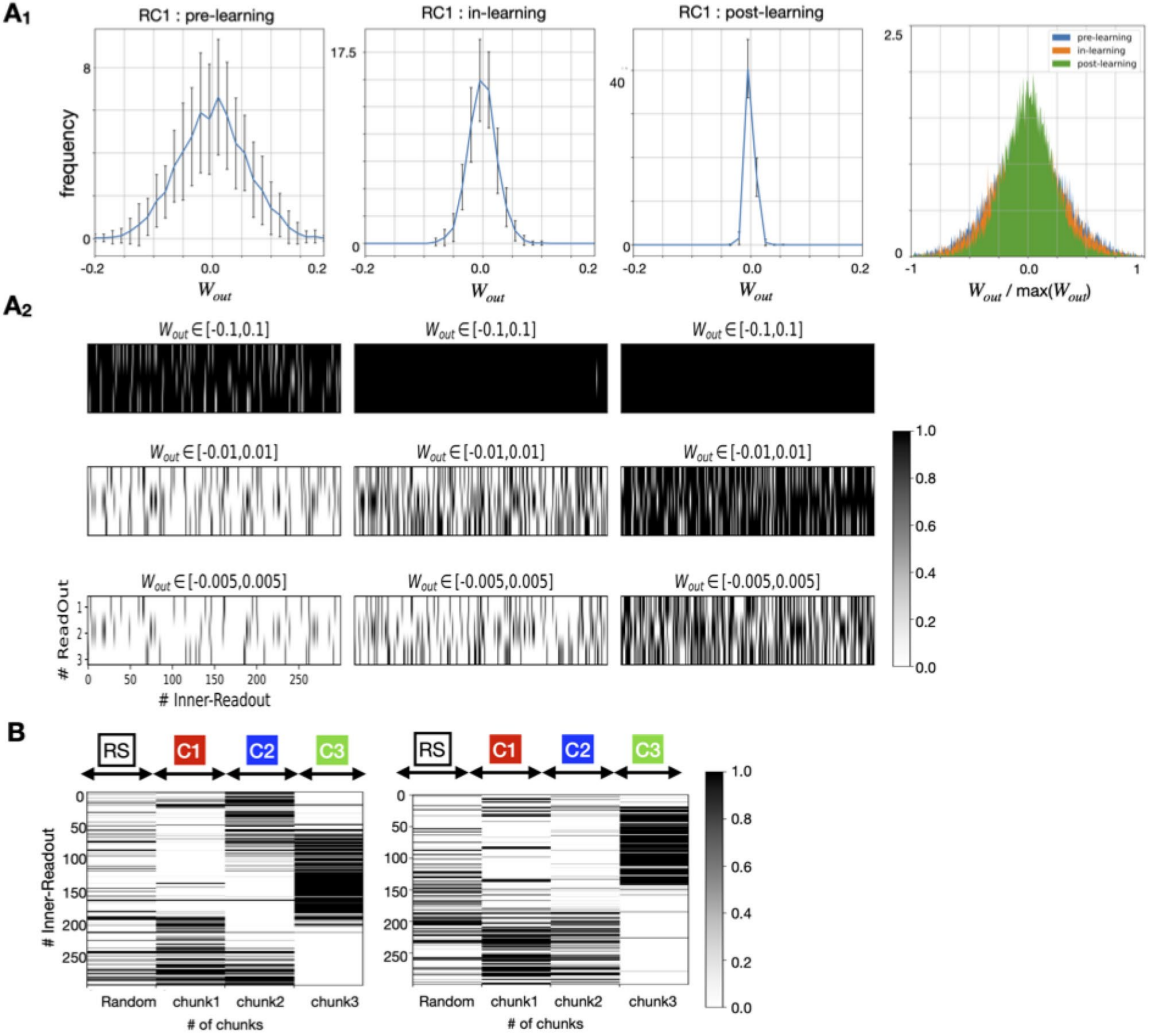
Evolution of reservoir-readout connections during and after learning. (**A**_**1**_) Time changes of distribution in the weight matrix connecting the reservoir internal unit to the readout unit. On the far right, a distribution of normalized connection magnitudes is shown for comparison with the distribution shapes across the stages of learning. The connections were normalized by the maximum value for each timing (**A**_**2**_). A detailed distribution of reservoir-readout connections is shown before, during, and after learning within the indicated range of ***W***^out^. White indicates that only a small portion of connections fall within a given range, whereas black indicates that many connections fall within a given range. (**B**) Selective decode responses to individual chunks (colored intervals) are self-organized. The input contains random sequences. The responses are gray-scaled according to their selectivity to the chunks. Left: the case where selectivity to a specific chunk by reservoir neuron projecting to readout units was clear. Right: the case where the selectivity was less clear.

Following learning, the norm of ${{\varvec{W}}}^{{\varvec{o}}{\varvec{u}}{\varvec{t}}}$ became smaller (Fig. 3A1 and A2), thus suggesting that a smaller portion of the units in the reservoir contributed to the readout activity after learning than before learning. As shown in Fig. 3B, the reservoir neurons projecting to readout units were organized such that those selectively respond to a chunk.

**Figure 4:**
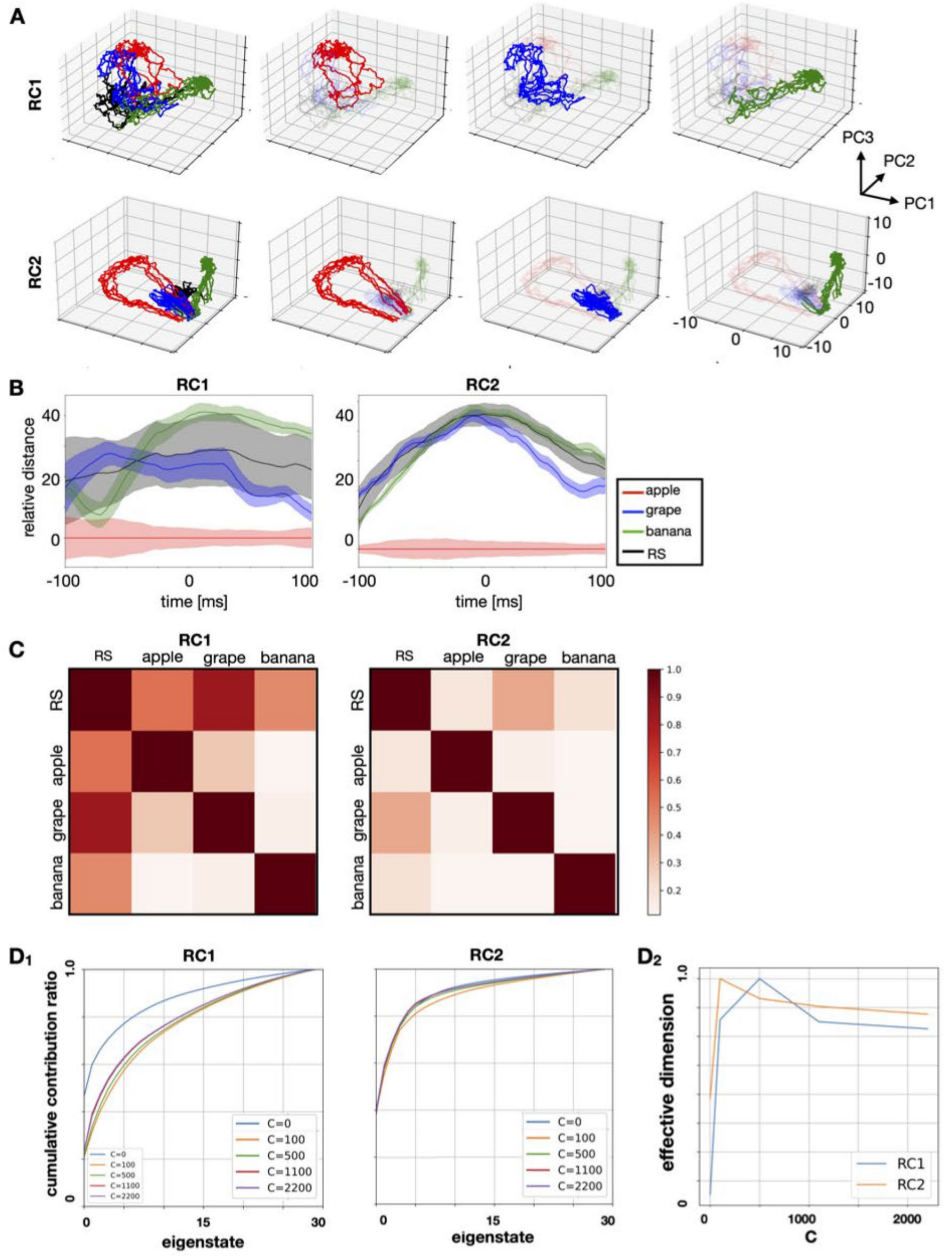
The characteristics of the trajectories of reservoir dynamics during stimulus presentation. (**A**) Results of projecting the activity inside the reservoir computing model for individual chunks (colored intervals) into the space from PC1 to PC3. Responses are self-organized for each chunk. The input contains a random sequence. Responses are color-coded according to their selectivity for chunks. Top: for the RC1 module. Bottom: for the RC2 module. (**B**) Distances between chunks of low-dimensional trajectories of reservoir dynamics. The distances ${d}_{XY}\left(t\right)$ were measured with respect to the “apple” trajectory (Y was set to “apple”). The shaded area indicates the deviation ${\sigma }^{\text{X}}(t)$. Because presentation times vary between chunks, the midpoint of the presentation time was set to 0, and the relative time from that point was used to display the data. (**C**) The degree of separation ${r}_{\text{XY}}$ between trajectories. The left and right sides indicate RC1 and RC2, respectively. The degree was defined in the Methods sections. The smaller the value is, the longer the distance between trajectories. (**D**_**1**_) Change in the cumulative contribution ratio before and during learning. The ratios for RC1 and RC2 are displayed on the left and right sides, respectively. (**D**_**2**_) Changes in the effective dimension trajectories for RC1 and RC2 after an increasing number of learning iterations. See the “Methods” section for the calculation of the effective dimension. *PC* principal component.

Next, to characterize the dynamics of reservoir networks during chunk presentation, we performed the principal component analysis to all reservoir units *N*. During each chunk presentation, the activity of the RC internal nodes drew a specific trajectory in the PC1 to PC3 space (Fig. 4A)^41–43^. The trajectories of reservoir neurons in RC1 (Fig. 4A top) and RC2 (bottom) appear different even for the same chunk owing to different connection configurations of reservoir networks and different time courses of inputs. Next, we examined the degree to which the trajectories corresponding to each chunk are separated from each other. Figure 4B displays the time course of relative distances between the "apple" trajectory and the other trajectories. Those trajectories were separated during the stimulus presentation without passing near the "apple" trajectory. The averaged distances between the trajectories show that the separation is sufficient (Fig. 4C). Thus, the trajectories were well distinguished between the chunks. It has been reported that population neural activity responsible for cognitive tasks, such as sensory perception, decision making, and motor control, is embedded in a lower dimensional subspace of the dimension *N* of the neural population^28^. To verify if the present model can achieve this feature, we characterized the dimension of the reservoir dynamics by calculating the accumulative contributions from the eigenstate with the largest eigen value. As shown in Fig. 4D1, the characteristics of the cumulative contribution of eigenstates differed between RC1 and RC2, presumably due to differences in temporal features between stimuli. We found a common tendency of increase in the dimensions immediately after learning. The trajectories were encoded with a low dimension for the network before learning (C = 0, which stored no information at all), yet as learning progressed, fewer eigenstates were needed for reconstruction, suggesting that the trajectories of reservoir networks were embedded within the lower-dimensional spaces. Although the effective dimension^44^ for the trajectory of RC1 was smaller than that of RC2 (Fig. 4D2), the gradual decrease of both these trajectories and the increase in the learning iteration C were consistent with the pattern seen in the cumulative contribution ratio (Fig. 4D1). The difference between trajectories in RC1 and RC2 was examined by varying the input parameters, and the results suggest that this difference depends on the timescale and gain of the input signal (Supplementary Materials, Fig. S1).

These results used the optimal parameter set, obtained by parameter exploration. The effect of different parameters on performance is summarized in the Supplementary Materials. The performance was found to depend on the time constant (*τ*) of reservoir neurons and the strength (*g*) of recurrent connections among reservoir neurons (Supplementary Figs. S2, S4). In addition to the model parameters, the timescale of the inputs was also found to affect the performance (Supplementary Figs. S2, S3, S4).

### Variations of interaction between sensory modules

**Figure 5:**
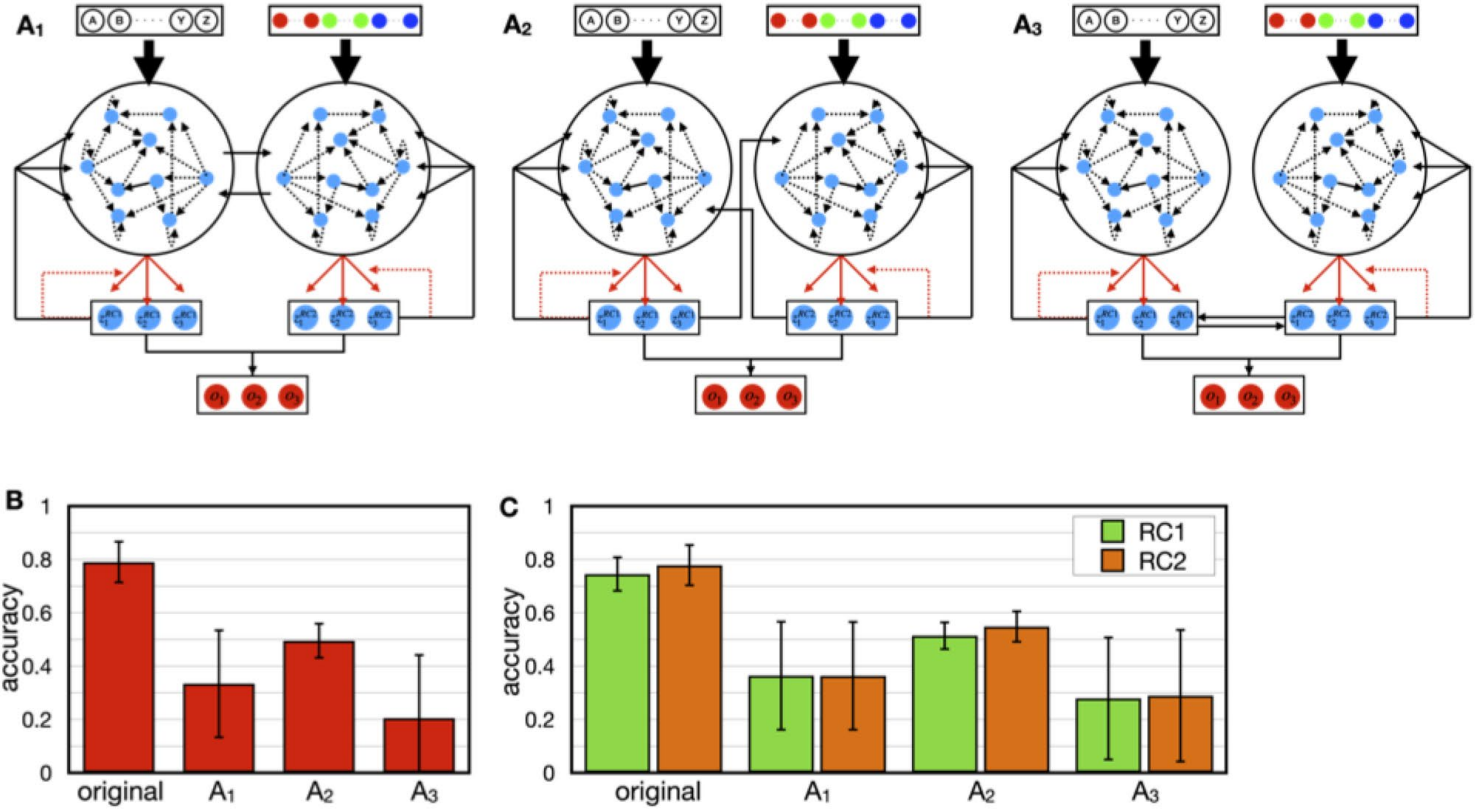
Variations of the model that have direct interactions between sensory modules. (**A**) Variations of ways of interaction between sensory RC modules. A_1_: The reservoir neurons in an RC module send connections to those in the other RC module. The connection matrices between the reservoirs ***W***^in, inter^ were determined in the same manner as ***W***^in^, but the probability of connections p was set to 0.1. A_2_: The readouts in an RC module feedback to reservoir neurons in the other RC module. The connection matrices of the inter-RC feedback ***W***^back, inter^ were determined in the same manner as ***W***^back^ with the same parameters as those for ***W***^back^. A_3_: The readouts in RC1 and RC2 were connected with each other. All-to-all connections determined the connectivity across RC modules. (**B**) Comparison of the accuracy between the original and alternative models A_1_ to A_3_. (**C**) The accuracy of the readout responses in RC1 (green) and RC2 (orange).

As shown above, the interactive RC model could segment paired sensory signals from continuous inputs and encode them as the dynamical state of sensory RC modules. In the model, the interaction between sensory modules is implemented in the teaching signal. Some studies have reported that interaction between different sensory information is achieved by direct connections between different sensory systems, as well as by top-down control from the higher systems, such as the association cortex^37–39^. For the interactive RC model, we could consider an alternative form of interaction between sensory modules when the readout neurons are interpreted as elements within the corresponding sensory module (Fig. 5A). In a possible form shown in Fig. 5A1, a portion of reservoir neurons in an RC module projects to those in the other RC module. This is the straightforward model of direct interaction between sensory systems. In another possible variation, the readout neurons feedback not only to reservoir neurons in the same module but also to the ones in the other module (Fig. 5A2). In the last model, the readout neurons of both modules are connected (Fig. 5A3). Unlike the original model, outputs of RC1 and RC2 are not necessarily associated in the models A_1_ to A_3_, to examine whether the association was essential for the readout’s learning. Therefore, the neurons in the association layer must be responsible for the associations between the outputs from RC1 and RC2. To do this, we introduced a simple learning rule based on the Hebb rule into the connections from readouts of RC modules to neurons in the association layer (see “Methods”) while, as in the original model, connections from reservoir neurons to readouts were trained by FORCE learning. Figure 5B shows a comparison between these alternative models and the original model (Fig. 1A) in terms of learning accuracy. Among these variations from A_1_ to A_3_, model A_2_ performed the best, but it was worse than the original model. With respect to the models A_1_ to A_3_, it is necessary that readouts of each RC module should show selectivity to the corresponding sensory modal input of a specific chunk. To confirm this, the accuracy at the level of RC modules was examined (Fig. 5C). These results indicate that alternative models did not percept the corresponding sensory modal inputs well. According to the result that Fig. 5C are similar to Fig. 5B, the low accuracy of models A_1_ to A_3_ can be attributed to the processing at RC modules, suggesting that the ways of interaction in those models should not be appropriate.

### The importance of co-occurrence matters

**Figure 6:**
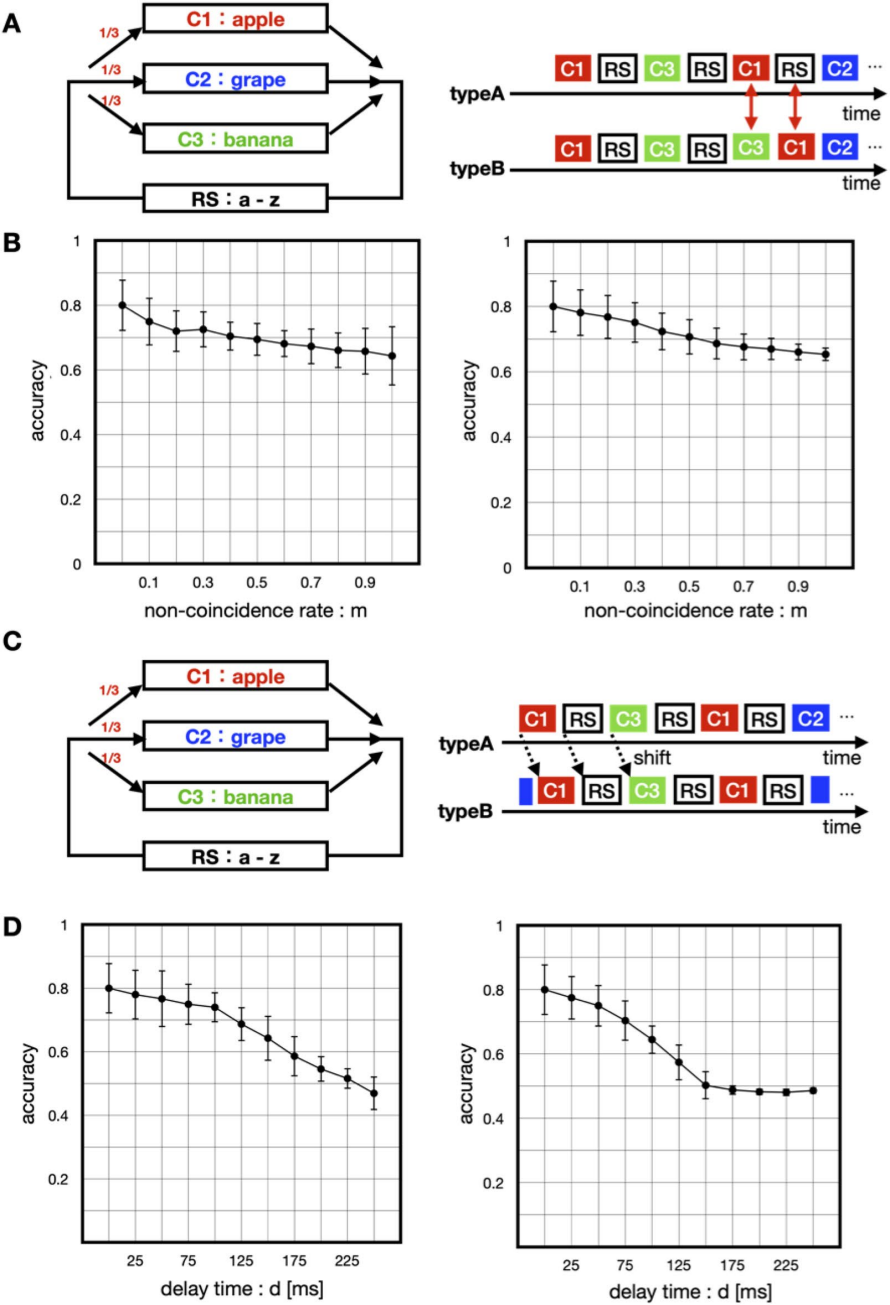
Reactions activated by non-contingent input through replacement and delay. (**A**) Three chunks of an apple (red), grape (blue), and banana (green) separated by random sequences recur at equal frequencies in the input. In non-co-occurrence signals, these chunks are swapped with a certain probability *m*. (**B**) Left: The relationship between non-coincidence *m* and the accuracy in training signals. Right: The relationship between non-coincidence* m* and the accuracy in testing signals. (**C**) Three chunks of an apple (red), grape (blue), and banana (green) separated by random sequences recur at equal frequencies in the input. In non-co-occurrence signals, these chunks are delayed with a certain time *d*. (**D**) Left: The relationship between the delay time d and the accuracy in training signals. Right: The relationship between the delay time *d* and the accuracy in testing signals.

The original model could recognize the co-occurrence of paired stimuli with different modalities. We demonstrated the model's response to inputs for missing paired stimuli and delayed stimuli. The co-occurrence of the corresponding stimuli in each modality was the key to activating the model. We divided the validation into a case where the model was trained with manipulated signals and the learning accuracy was verified with an unmanipulated input and a case where the manipulated signals were input to the model trained with unmanipulated signals in the validation phase. The accuracy was computed using ***o***(*t*) and the integrated output from the model. The missing input signal replaced one of the signals in a chunk pair with another signal or a random sequence, with a probability *m* (Fig. 6A). This probability varied from 0.0 to 1.0 in increments of 0.1. The correct response rate tended to decrease with an increase in the non-co-occurrence rate of the stimulus (Fig. 6B, left).

Next, we added a delay of time d to one of the signals, followed by its input (Fig. 6C). The delay time d varied from 0 to 250 in increments of 25 ms. Upon its input during learning, the accuracy decreased (Fig. 6D). Thus, in the case of missing signal input, the correct response rate tended to decrease with an increase in the inconsistency rate.

The aforementioned validation results indicated the importance of the temporal co-occurrence of specific stimulus patterns of each modality for model activation. In other words, the co-occurrence of specific information patterns in each heterogeneous modality was important for model activation and is key to information integration.

## Discussion

The brain is likely to associate with simultaneously presented visual and auditory stimuli. Repetitive simultaneous presentation of stimuli of different modes would promote the integration of the stimuli into a chunk by the brain to recognize the external event. This may be possible even in cases where simultaneous stimuli are not necessarily attributable to an identical event or object. Considering the importance of the temporal relationship between stimuli and the frequency of occurrence, we proposed a possible mechanism to integrate time-varying stimuli from different modalities. By examining how interactions between sensory modules affect multisensory integration, we found that the best performance (among the examined ways of interaction) was by the interaction of feedback from the outputs of appropriately learned sensory modules with alignment between sensory modules, suggesting that anatomical connectivity facilitates such an interaction between different sensory systems.

Given that an input activates the *i*-th readout in either RC module, the teaching signal is supposed to reinforce the *i*-th readout in the partner RC module by adjusting reservoir-readout connections. Thus, the input will be encoded by the *i*-th readouts of both RC modules. Considering that the brain possesses sensory module such as visual and auditory cortices and the modules directly interact each other, the interaction between RC modules might be the key for understanding the brain mechanism of multisensory integration. The earliest region where different sensory information converges is the superior colliculus (SC)^45,46^. Input from the association cortex assists the SC neurons in learning how to respond to multisensory inputs. In the proposed model, neurons in the association layer correspond to SC neurons, and connections between readout and association neurons should be regulated by the association cortex. However, the proposed model could not incorporate such learning because the association neurons are simplified and the higher layer was not included. In the computational model of multisensory integration based on such anatomical connectivity^47,48^, the unisensory modules that send sensory signals to SC neurons are designed to compete with each other in a winner-take-all fashion during the learning phase; this corresponds with the teaching signals employed in the proposed model.

The PCRC model has been proposed as a model to process time-varying sensory signals and encode them as the dynamical states of neural activity^36^. In the model with a similar structure to the present model, each sensory signal is encoded by the corresponding sensory RC such that the error between a sensory input and the RC output is minimized. Then, a multisensory signal is encoded by combining visual and auditory sensory information processed by the sensory RCs at the upper layer. Because the PCRC model uses predictive coding for encoding^49,50^, each sensory RC is trained such that it can generate the output that is the same as the given sensory input. Therefore, a sensory signal of a modality is essentially processed within the corresponding sensory module, while the proposed models interact with the other sensory module. (It should be noted that feedback from readouts is affected by the other sensory module, even in the original model.) If direct interaction between sensory modalities is to be considered, it may be necessary to redefine the essential mechanism; for example, what an RC module predicts is the input of a single modality or integrated inputs of related sensory modalities. On the other hand, the interaction between modalities is incorporated in the proposed model, although it is designed as the teaching signal. Furthermore, the model can be transformed as shown in Fig. 6, allowing us to evaluate how the interactions should be, although we need to find a better learning mechanism. From the viewpoint of the learning principle, it is essential in reservoir computing to project an input into a higher dimensional complex time-series and to construct the desired output from the time-series. In the basic form of reservoir computing, these two processes can be performed simultaneously by training connections from reservoir units to readouts. While the former is achieved in both the PCRC and the proposed model, the latter is different between these two models. In the PCRC model, these processes are achieved so that the output of readouts can reproduce inputs by using existing inputs as teaching signals. Therefore, to construct outputs for a higher-level structure such as the integration module, separate processing is required. The proposed model avoids this by using outputs from the other module as teaching signals.

The co-occurrence of stimuli of different modes would be attributable to an identical cause, particularly in the case of frequent presentation. The proposed model was capable of integrating simultaneously presented paired stimuli, and its performance deteriorates with decreased probability of co-occurrence or an increased delay of the presentation of one of the paired stimuli (Fig. 6). Concerning the influence of the delay on multisensory integration, asynchronous presentation (i.e., with the delay) causes weak integration^51^. Perception by multimodal integration decreased as the delay became longer. The percentage of correct perception drops to 50% when the delay increases to 200–300 ms^3^. Although the accuracy shown in Fig. 6D is not necessarily comparable to the correct perception percentage, the model showed a similar dependence on the delay. However, the effect of the auditory and visual stimuli on the perception is asymmetric; for example, pairs of visual and auditory stimuli in which the visual one precedes the auditory one are sometimes recognized as a synchronous stimulus pair^3^. Furthermore, such perception highly depends on stimulus types (such as flash and beep, a tool and its sound, and speech with video), suggesting that a higher cortical area may be involved in the process. This property would be difficult to reproduce within the present model because the top-down control that would arise from such a higher structure is not represented in the present model.

The probability of co-occurrence of paired stimuli and the temporal delay between these stimuli displayed a strong association with the “McGurk effect”^3^ and “ventriloquism effect”^52^; the “McGurk effect” is a phenomenon in which an unfamiliar combination of sensory information, each of which is familiar, leads to the new perception, and “ventriloquism effect” is the auditory illusion that sound is perceived as coming from the different location, but not from the location where the sound is actually generated owing to visual stimuli. Both are major issues in multisensory integration because the dependence of perception on temporal aspects of the stimuli would imply that the integration process depends on dynamic shifts in properties of neural activity; the chunking mechanism based on neural population dynamics may also be involved in the illusional perception. The “McGurk effect” can be resolved by treating the multimodal information as a static representation of a joint probability distribution representing combined stimuli of different modalities^53,54^; however, it is impossible to treat the “ventriloquism effect” by such representation because it does not incorporate the time course of sensory signals. In particular, it is reported that the “ventriloquism effect” is affected by the temporal difference between visual and auditory stimuli and the symmetrical/asymmetrical effect between visual and auditory stimuli exits^52^. The present model would enable us to consider the effect of the temporal difference or how to interact between the modules. In the ventriloquism effect, when there is a mismatch between the location of auditory and visual stimuli, a calibration occurs in which the perception of the location of auditory stimulus is drawn to the location of visual presentation. Traditionally, it has been assumed that repeated experience of such mismatch stimuli is required for this calibration to be achieved, but one study found it to occur after only the immediately preceding trial^55^. Extensions of this model that can encode stimulus location may allow us to test whether such short-term calibration can occur via a bottom-up mechanism, such as memory retention by recurrent dynamics. Therefore, the proposed model may contribute to a unified understanding of how temporal characteristics affect multisensory integration efforts beyond the McGurk and ventriloquism effects.

In the proposed model, neurons in the higher association layer were modelled to encode the results of multimodal integration by responding to specific co-occurrences of paired stimuli. This layer should be modelled to integrate multimodal information from the lower layers in a self-organizing manner, which would enable each neuron in this layer to display its response to specific combinations of multisensory information among numerous combinations. This modified version could be tested as a possible underlying mechanism of the “McGurk effect.” For example, the model could be validated by evidence showing that the degree of the “McGurk effect” depends on the temporal order and intervals of auditory and visual stimuli^2^. In our model, the interaction between the different modal modules (RC1 and RC2) was direct. The interaction via a higher layer may be possible because perception requires top-down control from higher-order brain areas, such as the association cortices, whereas direct connections between primary sensory areas allow those areas to interact with each other. In the ventriloquism effect, the influence of visual information on auditory information could be stronger than vice-versa, although either is influenced by the accuracy of modal information.^56,57^. It is important to determine whether the above-mentioned asymmetric result is caused by the characteristics of bottom-up processing (which may arise from asymmetry between processing of different sensory signals), by an asymmetric interaction between different sensory modules, or by asymmetric top-down control^56–61^.

## Methods

### Details of the network models

In this study, we adopted a collaborative RC model that was originally developed to extract meaningful segments from information streams. The model consisted of two recurrent networks called echo state networks (ESNs)^62^, each of which has three layers: the input, reservoir, and output. The reservoir layer is a recurrent neural network that consists of *N* rate-based neurons. A neuron in the reservoir layer obeys the following dynamics:

4 $$\uptau \frac{d{\varvec{x}}}{dt}=-{\varvec{x}}\left(t\right)+g{\varvec{W}}{\varvec{r}}\left(t\right)+{{\varvec{W}}}^{\text{in}}{\varvec{i}}\left(t\right)+{{\varvec{W}}}^{\text{back}}{\varvec{z}}\left(t\right)+k{\varvec{\upxi}}\left(t\right),$$

5 $${r}_{i}\left(t\right)=\mathrm{tanh}\left({x}_{i}\left(t\right)\right),$$

6 $${\varvec{z}}\left(t\right)={{\varvec{W}}}^{\text{out}}{\varvec{r}}\left(t\right),$$

where ***i***(*t*) is the activity of the neuron in the input layer, *g* is the parameter that scales connections between neurons in the reservoir layer and controls the complexity of the network behavior; the reservoir network shows chaotic spontaneous activity for *g* > 1. ***W***, ***W***^in^, and ***W***^back^ are connections within reservoir neurons, those from the input to the reservoir layer, and those from the reservoir to output layers, respectively. ***r***(*t*) and ***W***^out^ are the output of the reservoir neurons, and connection matrix from the reservoir neurons to output neurons that is also called the readout weight matrix, respectively. Each output neuron receives inputs from *S* reservoir neurons by the weights ***W***^out^. The output neurons projected back to all reservoir neurons. Learning was conducted by modifying the readout weight matrix ***W***^out^ based on the FORCE algorithm that minimizes the error between the output and the teaching signal^40^. The elements of ***W*** are generated by a Gaussian distribution of *N*(0, 1/(*pN*)) where p is the probability of connections between reservoir neurons. The elements of ***W***^in^ were determined such that each row had only one non-zero element generated by a normal distribution *N*(0,1). Elements of ***W***^back^ were drawn from a uniform distribution [-1, 1]. The initial values of Wout are generated by a Gaussian distribution *N*(0, 1/*N*). Table 1 summarizes parameter values. The time step of the simulation is 1 ms. All experiments were performed 60 times^31,63^.

**Table 1:** A list of model parameters used in the validation.

	Basic	Supplementary Figs. S2A–C, S5A, B	Supplementary Fig. S2E	Supplementary Fig. S5C	Supplementary Fig. S5D	Figure 6B	Figure 6D
m	0	0	0	0	0	0–1 in 0.1 increments	0
d	0	0	0	0	0	0	0–250 in 25 increments
C	2200	2200	2200	2200	100–5000 in 100 increments	2200	2200
α	100	100	100	100	100	100	100
β	3	3	3	3	3	3	3
γ	0.5	0.5	0.5	0.5	0.5	0.5	0.5
δ	0.5	0.5	0.5	0.5	0.5	0.5	0.5
I^RC1^	26	26	26	26	26	26	26
I^RC2^	30	30	30	30	30	30	30
O	3	3	3	3	3	3	3
N	1200	600–1200 in 200 increments	1200	1200	1200	1200	1200
S	300	100–600 in 100 increments	300	300	300	300	300
p	0.5	0.5	0.5	0–1 in 0.1 increments	0.5	0.5	0.5
τ	5	5–35 in 10 increments	5	5	5	5	5
g	1.0	1.0	0–2 in 0.1 increments	1.0	1.0	1.0	1.0
k	0.2	0.2	0 or 0.2	0.2	0.2	0.2	0.2

Parameters used in simulations are as follows: *m, d*, and *C* are parameters related to the input signal, where *m* is the non-coincidence rate of the input stimulus, *d* is the delay time, and *C* is the total number of stimuli, including random sequences. *α, β, γ*, and *δ* are parameters involved in the learning of the network. *α* plays a role in the learning rate in FORCE learning. *β*, *γ*, and *δ* are constants. For more information on *α*, see Sussillo^40^. *I*^*RC1*^*, I*^*RC2*^*, O, N*, and *S* are the parameters that make up an RC. *I*^*RC1*^ and *I*^*RC2*^ are the numbers of input nodes, *O* is the number of read nodes, *N* is the number of reservoir nodes, and *S* is the number of reservoir nodes coupled to the read nodes of RC1 and RC2. *p, τ, g*, and *k* are the parameters that control the dynamics of the reservoir neurons. *p* is the connection probability of reservoir neurons, *τ* is the time constant that determines the dynamics of the reservoir neurons, *g* is the parameter that controls the dynamics, and *k* is the rate at which noise is introduced.

### Teaching signals

In the learning rule, we normalized the outputs such that the mean and the standard deviation of outputs were satisfied with 0 and unity, respectively:

7 $${\varvec{z}}\left(t\right)\to \widehat{{\varvec{z}}}\left(t\right)=\frac{z\left(t\right)-{\varvec{\upmu}}\left(t\right)}{{\varvec{\upsigma}}\left(t\right)},$$

where ${\varvec{\upmu}}\left(t\right)$ and ${\varvec{\upsigma}}\left(t\right)$ were calculated as follows:

8 $${\varvec{\mu}}\left(t\right)=\frac{1}{T}{\int }_{t-T}^{t}{\varvec{z}}\left({t}^{^{\prime}}\right)d{t}^{^{\prime}},$$

9 $$\upsigma \left(t\right)=\sqrt{\frac{1}{T}{\int }_{t-T}^{t}{z}^{2}\left({t}^{^{\prime}}\right)d{t}^{^{\prime}}-\upmu {\left(t\right)}^{2}},$$

with a sufficiently long period *T* = 15,000 ms.

The teacher signal for the proposed model was determined using the following equation:

10 $${f}_{i}^{{\text{RC}}1}\left(t\right)={\left[\mathrm{tanh}\left(\frac{{\widehat{z}}_{i}^{RC2}\left(t\right)-\upgamma \left({\sum }_{j}{\widehat{z}}_{j}^{RC2}-{\widehat{z}}_{i}^{RC2}\right)}{\upbeta }\right)\right]}_{+},\left(i,j=\mathrm{1,2},3\right),$$

11 $${e}_{i}^{RC1}\left(t\right)= {z}_{i}^{RC1}\left(t\right)- {f}_{i}^{RC1}\left(t\right),$$

where ${\widehat{z}}_{i}\left(t\right)$ is the *i*-th normalized output. The threshold linear function [*x*]_+_ returns 0 for *x* ≤ 0 and *x* for *x* > 0; therefore, the output value was positive. The numerator inside the function tanh() takes a positive value if the *i*-th readout showed the largest response because the second term of the numerator represents an average except the *i*-th readout. Therefore, when the *i*-th readout of an RC module responded to a chunk the most, the teacher signal reinforces the *i*-th readout of the other RC module. Each output neuron in an RC module received the teacher signal defined by Eq. (10) from the partner RC module. The signal exerted a collaborative or competitive influence depending on the chunk presentation. The type of chunk an output neuron should learn was not assigned in advance, and the model was self-organized based on the teacher signals. Because the teacher signal is symmetric concerning the permutation of the index, output neurons with the same index responded to the same chunk. In the results, output neurons in both RC modules showed responses similar to each other. Usage of the tanh function limited output ranges and prevented the model from responding to a specific chunk too much. *β* and *γ* were set to 3 and 0.5, respectively. The error between the teacher signal and the output was defined using Eq. (11).

### Designed output

From the symmetry of the teacher signals for RC1 and RC2, ${z}_{i}^{{\text{RC}}1}$ and ${z}_{i}^{RC2}$ react to a similar chunk. However, multimodal information may lead to varied response profiles of the readout units for RC1 and RC2. We should integrate the RC1 and RC2 outputs to calculate the accuracy rate of the model. Therefore, we used *o*_*i*_(*t*) for the final performance comparison of the model. We set the constants to *δ* = 0.5.

12 $${o}_{i}\left(t\right)=\delta \left({z}_{i}^{{\text{RC}}1}\left(t\right)+{z}_{i}^{{\text{RC}}2}\left(t\right)\right).$$

In the alternative models presented in Fig. 6A1, A2, and A3, the teacher signal is closed in the own RC modules unlike the original model. Therefore, readouts between RC1 and RC2 do not always correspond. For example, the *i*-the readout in the RC1 is responsible for the chunk “apple”, while the *j*-th readout in the RC2 may respond to the chunk “apple”. The neurons in the association layer must organize the outputs of RC1 and RC2 so that each neuron in the layer represents one of the learned chunks. For this purpose, we introduced the learning rule into connections from the readouts of RC1 and RC2. Let ${w}_{ij}^{x}$ be the connection from the *j*-th readout of RC*x* to the *i*-th neuron in the association layer. The output of *o*_*i*_*(t)* and the dynamics ${w}_{ij}^{x}$ is defined as

13 $${o}_{i}\left(t\right)={\sum }_{j}^{N}{w}_{ij}^{{\text{RC}}1}{z}_{j}^{{\text{RC}}1}(t)+{\sum }_{j}^{N}{w}_{ij}^{{\text{RC}}2}{z}_{j}^{{\text{RC}}2}(t),$$

14 $$\frac{d{w}_{ij}^{x}}{dt}={o}_{i}\left(t\right){z}_{j}^{{\text{RC}}x}\left(t\right)-{o}_{i}\left(t\right)\left(\sum_{j}{z}_{j}^{{\text{RC}}x}\left(t\right)\right)/N,$$

where *N* is the number of readouts or neurons in the association layer (*N* = 3). The first and second term of the right-hand side represents the Hebbian rule and the normalization, respectively.

### Evaluation method

Let *o*(*t*) be the output to be analyzed. Convolution of *o*(*t*) with the window function *w*(*t*): ${\int }^{t}w\left(\tau \right)o\left(\tau -t\right)d\tau$ was taken. For the window function, the Gaussian function was used. Then the value of convoluted output was normalized within the range of 0 and 1. The normalized time course of the convoluted output was segmented into periods of chunk presentation. For each period, the unit showing the largest response was determined. After this process, we found which output unit responded to each chunk presentation for all chunk presentation. According to which chunk an output unit most responded to, we determined the relationship between output units and chunks. We should note that “no responses” of all output neurons were regarded as the correct response to random sequences. Based on this result, we could obtain a confusion matrix: each row of the matrix represents a chunk, whereas each column represents an output unit. An element of the matrix includes the number of counts that the corresponding unit (column) responded to the corresponding chunk (row). If the indices of output units were sorted based on the indices of chunks that each unit was related to, then diagonal elements contain the counts of correct responses, which represent true-positives for binary discrimination.

According to the obtained confusion matrix, the accuracy rate was calculated as follows:

15 $$accuracy=\frac{{\sum }_{i}{c}_{ii}}{C},$$

where *C* represents the total number of stimulus presentation epochs, including random sequences (the sum of the counts in all boxes of the matrix). *c*_*ij*_ represents the element in the *i*-th row and the *j*-th column. Therefore, the numerator is the sum of diagonal elements, indicating the sum of correct responses for all presentations. Considering learning was unsupervised, the stimulus selectivity was not explicitly imposed on each unit. Therefore, we determined the assignment of chunk selectivity for each unit such that the accuracy could be maximized.

### Activities of the reservoir unit

To define the response selectivity of reservoir neurons, we sorted them based on their mean activation phases:

16 $$\overline{{t }_{i}}\left(t\right)=\frac{T}{\uppi }arg\left[\frac{{\sum }_{{t}^{^{\prime}}=1}^{T}\overline{r }\left({t}^{^{\prime}}\right)\mathrm{exp}\left(i\frac{2\pi {t}^{^{\prime}}}{T}\right)}{{\sum }_{{t}^{^{\prime}}=1}^{T}\widehat{{r}_{i}}\left({t}^{^{\prime}}\right)}\right],$$

where $\overline{{\varvec{r}} }\left(t\right)$ is the normalized output of a reservoir neuron. Each reservoir neuron exhibited a phasic response to a specific chunk, which indicated its selectivity. We considered only the reservoir neurons projecting to output neurons.

### Calculation of relative distance between trajectories

A state of an RC module in low-dimensional space spanning from PC1 to PC3 at *t* when an input X is presented for the *i*-th trial is denoted by ${{\varvec{x}}}_{\text{X}}^{i}\left(t\right)=({x}_{\text{X}}^{i}\left(t\right), {y}_{\text{X}}^{i}\left(t\right), {z}_{\text{X}}^{i}\left(t\right))$ where X = apple, grape, or banana. The point at *t* averaged over trials, which is denoted as the center point at *t* for input X, is obtained as $\overline{{{\varvec{x}} }_{\text{X}}}\left(t\right)=(\overline{{x }_{\text{X}}}\left(t\right), \overline{{y }_{\text{X}}}\left(t\right), \overline{{z }_{\text{X}}}\left(t\right))$, where $\overline{{x }_{\text{X}}}\left(t\right)=\frac{1}{{n}_{\text{X}}}\sum_{i=1}^{{n}_{\text{X}}}{x}_{\text{X}}^{i}\left(t\right), \overline{{y }_{\text{X}}}\left(t\right)=\frac{1}{{n}_{\text{X}}}\sum_{i=1}^{{n}_{\text{X}}}{y}_{\text{X}}^{i}\left(t\right), \overline{{z }_{\text{X}}}\left(t\right)=\frac{1}{{n}_{\text{X}}}\sum_{i=1}^{{n}_{\text{X}}}{z}_{\text{X}}^{i}\left(t\right)$ *A*nd *n*_X_ is the number of trials for input X.

Thus, the distance between the center points of input X and Y, ${d}_{\text{XY}}(t)$, is obtained by ${d}_{\text{XY}}\left(t\right)=\left|\overline{{{\varvec{x}} }_{\text{X}}}\left(t\right)-\overline{{{\varvec{x}} }_{\text{Y}}}\left(t\right)\right|$, where | | denotes the Euclidean norm. The variance in the direction of PC1 ${{\sigma }_{x}^{\text{X}}(t)}^{2}$ is ${{\sigma }_{x}^{\text{X}}(t)}^{2}=\frac{1}{{n}_{\text{X}}}{\sum }_{i=1}^{{n}_{\text{X}}}{({x}_{X}^{i}\left(t\right)-\overline{{x }_{\text{X}}(t)})}^{2}$. The deviation of the trajectories X around the center point at *t* is defined as ${\sigma }^{\text{X}}\left(t\right)=\sqrt{{{\sigma }_{x}^{\text{X}}(t)}^{2}+{{\sigma }_{y}^{\text{X}}(t)}^{2}+{{\sigma }_{z}^{\text{X}}(t)}^{2}}$.

Therefore, we defined the degree of separation between trajectories of input X and Y as ${r}_{\text{XY}}$, where $r_{{{\text{XY}}}} = \sum _{{s = t - 100}}^{{t + 100}} \frac{{\sigma ^{X} \left( s \right) + \sigma ^{Y} (s)}}{{d_{{{\text{XY}}}} (s)}}$ .The trajectories have left transient states at the midpoint of presentation times *t* = 0. Therefore, we decided to characterize it by cutting out the 100 steps before and after t = 0 and integrating the time points.

The smaller the value of ${r}_{\text{XY}}$ is, the more separated the two trajectories are.

### Effective dimension

Effective dimension is a measure of the effective number of principal components describing a trajectory^44^. For example, in the effective dimension, if *n* principal components equally share the total variance and the remaining *N* principal components have zero variance, then ${N}_{eff}=n$.

The effective dimension is given by

$${N}_{eff}={\left(\sum_{a=1}^{N}{\lambda }_{a}^{2}\right)}^{-1},$$

where ${\lambda }_{a}^{2}$ is the eigenvalue.

## Supplementary Information

Supplementary Information.

## Data Availability

The source code (Python code) is available at https://zenodo.org/record/5835322#.Yd0hUi2MuP0.
