# Supplementary material for: Association between different sensory modalities based on concurrent time series data obtained by a collaborative reservoir computing model
Source: Sci Rep. 2023 Jan 4;13:173. doi: 10.1038/s41598-023-27385-x (PMC9813012; doi:10.1038/s41598-023-27385-x)

**SUPPLEMENTARY MATERIALS**

**Dimension of reservoir dynamics**

As shown in Fig. 4A, D_1_, and D_2_, dimension trajectories RC1 and RC2 differed from each other. Therefore, we examined the effect of the timescale and gain of input on these trajectories. As shown in Figure S1A, we found that the effective dimension of the reservoir trajectory slightly decreased with the longer timescales, and that the gain had a significant impact on the dimension; specifically, that the dimension markedly decreased with the larger gains. This tendency is confirmed by examples of trajectories in Fig. S1B (timescale = 200, gain = 1.0) and Fig. S1C (timescale = 200, gain = 3.0). In comparison with image inputs (Fig. 1C), the text tone inputs can be regarded as a signal with a shorter timescale and larger gain. Thus, this result suggests that the timescale of gain of the input signal has a significant impact on the reservoir dynamics and their dimensions.

**Fig S1: Dimension of reservoir dynamics**

(A) Dependence of the effective dimension on the timescale and gain of an input. The dimensions of reservoir dynamics in RC1 (left) and RC2 (right) are shown. (B) The trajectories when the timescale and gain are set to 200 (ms) and 1.0, respectively. The circles, the squares, and the triangles indicate the states at the stimulus onset, at the midpoint of stimulus, and at the end of stimulus, respectively. (C) Similar to (B), but with the gain set to 3.0. The symbols are the same as (B).

**Dependence of the performance on model parameters**

Learning performance would depend on the properties of the model that is determined by model parameters. In particular, the parameters concerning the dynamical properties would influence the performance for processing time-varying inputs. Herein, we investigated the dependence of the correct answer rate on the model parameters to determine the dynamical properties of the model. The time constant of reservoir neurons might be an essential parameter for the dynamics. Therefore, we examined the time constants *τ* (5, 15, 25, and 35). The accuracy was high for *τ*=5 (Fig. S2A). Rapid replacement of the state inside the reservoir with another state would improve the accuracy. In other words, the recurrent network stored information only for a brief period. In this investigation (Fig. S2A), the time constants of RC1 and 2 were set to the same values. Because the time courses on inputs are different between text tone and image inputs, the optimal value of the time constant may differ between the RC modules. Therefore, in further investigations (Fig. S2B and C), the constant for RC1 (RC2) was varied, and that of the other RC module was kept constant. As shown in the figures, the dependence of the learning accuracy on the time constant was found small, although the time constant for RC1 was better for approximately 25‒30 ms, whereas the smaller value was better for RC2. Additionally, when the timescale of input changes, whether the learning performance depends on the time constant is examined (Fig. S2D). In the case where the input timescale is relatively small (25–50 ms), the time constant of reservoir neurons should be smaller for better learning accuracy. When the timescale is larger (>100 ms); however, the learning accuracies for different time constants become smaller. In other words, while a small time constant produces good performance for any input timescale, a large time constant performs well only for a large input timescale. If elongated and shortened versions of learned inputs were given, the model could not respond to all the inputs correctly (Fig. S3), suggesting that the model did not show time invariance as reported^2^. In accordance with the previous model^1^, connections in the reservoir networks were configured to realize the edge of chaos^3,4^ by setting the scaling parameter g to 1.0. However, it has been reported that the addition of noise causes the system to leave the regime on the edge of chaos^5^. To realize the edge of chaos under the noise-added condition, the parameter should be set to a larger value. Therefore, we compared the learning performance using a value ranging from 0.0 to 2.0 (Fig. S2E). As indicated, the magnitude of *g* had a significant effect on performance in the noiseless condition (*k* = 0), whereas it was found to have a weaker effect on performance in the noise condition. Since performance did not significantly deteriorate when *g* = 0 (no recurrent connections), we further examined how the system functions in such a condition. When *g* = 0.0, the reservoir neuron dynamics Eq. 6 is as follows

$\tau\frac{d\boldsymbol{x}}{dt}=-\boldsymbol{x}\left( t \right)+\boldsymbol{W}^{\text{in}}\boldsymbol{i}\left( t \right)+\boldsymbol{W}^{\text{back}}\boldsymbol{z}\left( t \right)+k\boldsymbol{\xi}\left( t \right)$.

If we substitute Eq. 7 and 8 into ***z***(*t*), the above equation turns to be

$\tau\frac{d\boldsymbol{x}}{dt}=-\boldsymbol{x}\left( t \right)+\boldsymbol{W}^{\text{in}}\boldsymbol{i}\left( t \right)+\boldsymbol{W}^{\text{back}}\boldsymbol{W}^{\text{out}}\boldsymbol{r}\left( t \right)+k\boldsymbol{\xi}\left( t \right) \text{and} r_{i}\left( t \right)=tanh(x_{i}\left( t \right))$.

The reservoir neurons have no direct mutual connections, but those have indirect mutual connections through the readout neurons (the third term in the right-hand side). Further, we set ***W***^back^ = **0** to remove the “indirect” mutual connections, the dynamics is as follows,

$\tau\frac{d\boldsymbol{x}}{dt}=-\boldsymbol{x}\left( t \right)+\boldsymbol{W}^{\text{in}}\boldsymbol{i}\left( t \right)++k\boldsymbol{\xi}\left( t \right)$.

The inputs were given to this feedforward network to determine its mechanisms. We examined the following three cases: pairs of text tones and an image one auditory (RC1) module and to one visual (RC2) (the same as in the default setup; Fig. S4A), image inputs to both modules (Fig. S4B), and text tones to both modules (Fig. S4C). The outputs of neurons in the “association” layer *o*(t) seems to respond appropriately in the former two cases. In contrast, the outputs looked much different in the last case. We then input the texts in the reverse order (e.g., “elppa”) to the network after learning the texts in the correct order (e.g., “apple”). Fig. S4D indicates that the response order of the “association” neurons to the reversed input is opposite (right) to that of the correct input (left), suggesting that those neurons might be responding to a letter (in the example, “a”), but not to a chunk. These different results can be attributable to difference in time course of inputs. As shown in Fig. 1B and C, the text tone input was discontinuous when viewed from each input node, while image input was continuous. In the other words, while the autocorrelation of text tone input is low, the autocorrelation of image input is larger, which suggests that input itself contains “memory.” This is presumably because the network performs well for image inputs. In the case of the default setup (the first case), the output of RC2, which represents image inputs, complement with the poor responses of RC1 output, which represents auditory inputs. Thus, we can conclude recurrent connection within a reservoir layer is necessary for the general input.

With respect to the other parameters, we investigated number of reservoir units *N,* number of reservoir units connected to the readout unit *S*, connection probability between reservoir neurons *p*, and number of iterations in the learning phase *C* because these parameters would have an influence on the behavior of the system and its performance. Those results are summarized in Fig. S5. Further investigation was performed for the role of noise *k* in the reservoir units, and the nonlinearity of the teacher signal *β*. This tendency was similar to that previously reported (data not shown)^1^. Taken together, the best number of reservoir units was *N*=1200, which was highly accurate, thereby suggesting that a larger network did not always improve performance. In other words, the learning performance was not necessarily proportional to the network size.

**Fig S2: Dependence of the accuracy on parameters**

(A) Accuracy changes when the time constant of reservoir neurons is changed. (B) Accuracy changes when the time constant of reservoir neurons in RC1 and the time constant for RC2 was set to 5 ms. (C) similar to (B), but when the time constant for RC2 was changed. (D) Accuracy changes when the timescale of text tone inputs was changed for various time constants of reservoir neurons. The timescale is defined by the decay time constant of a text tone. (E) Dependence of the accuracy on *g* with/without noise.

**Fig S3: Response to elongated and shortened inputs**

(A) Responses to the learned input after learning. The timescale of inputs was set to 50 ms. (B) Responses to shortened inputs. After learning inputs used in (A), the shortened version of the inputs was inputted to the model. The timescale was set to 25 ms. (B) Responses to elongated inputs. Similar to (B), but the elongated version of inputs was given. The timescale was set to 150 ms.

**Fig S4: Response of the network without recurrent connections within a reservoir layer**

(A) Responses of readout neurons in RC1 (top), RC2 (middle) and neurons in the “association” layer (bottom) when pairs of a text tone input and an image input were given to RC1 and RC2, respectively. (B) Similar to (A), but when pairs of image inputs were given to both RC1 and RC2. (C) Similar to (A), but when pairs of text tone inputs were given to both RC1 and RC2. (D) The response to the input in the correct order (left) and in the reversed order (right) after learning pairs of text tone inputs in the correct order. The red arrows indicate the epoch shown in (C).

**Fig. S5: Dependence of learning performance on model parameters**

(A) Accuracy changes when the number of reservoir units *N*=600, 800, 1000, and 1200 is changed with *τ*= 5, 15, 25, and 35. (B) Accuracy changes when the number of reservoir units projecting to readout units *S*=100, 200, 300, 400, 500, and 600. (C) Dependence of performance on connection probability within reservoir neurons *p*. (D) Network performance depends on the numbers of repetitive chunks in the input, separated by random sequences. (E) Relationship between the probability of chunk appearance and accuracy.

**References**

1. Asabuki, T., Hiratani, N., & Fukai, T. Interactive reservoir computing for chunking information streams. *PLoS Comput Biol.* 14, e1006400 (2018).

2. Goudar, V., & Buonomano, D.V. Encoding sensory and motor patterns as time-invariant trajectories in recurrent neural network. *Elife* 7, e31134.

3. Legenstein, R. & Maas, W. Edge of chaos and prediction of computational performance for neural circuit models. *Neural Netw.* 20, 323-334 (2007).

4. Boedecker, J. Obst, O. Lizier, J.T. Mayer, N.M. Asada, M. Information processing in echo state networks at the edge of chaos. *Theory Biosci.* 131, 205-213 (2012).

5. Massar, M. & Massar, S. Mean-field theory of echo state networks. *Phys. Rev. E* 87, 042809 (2013).


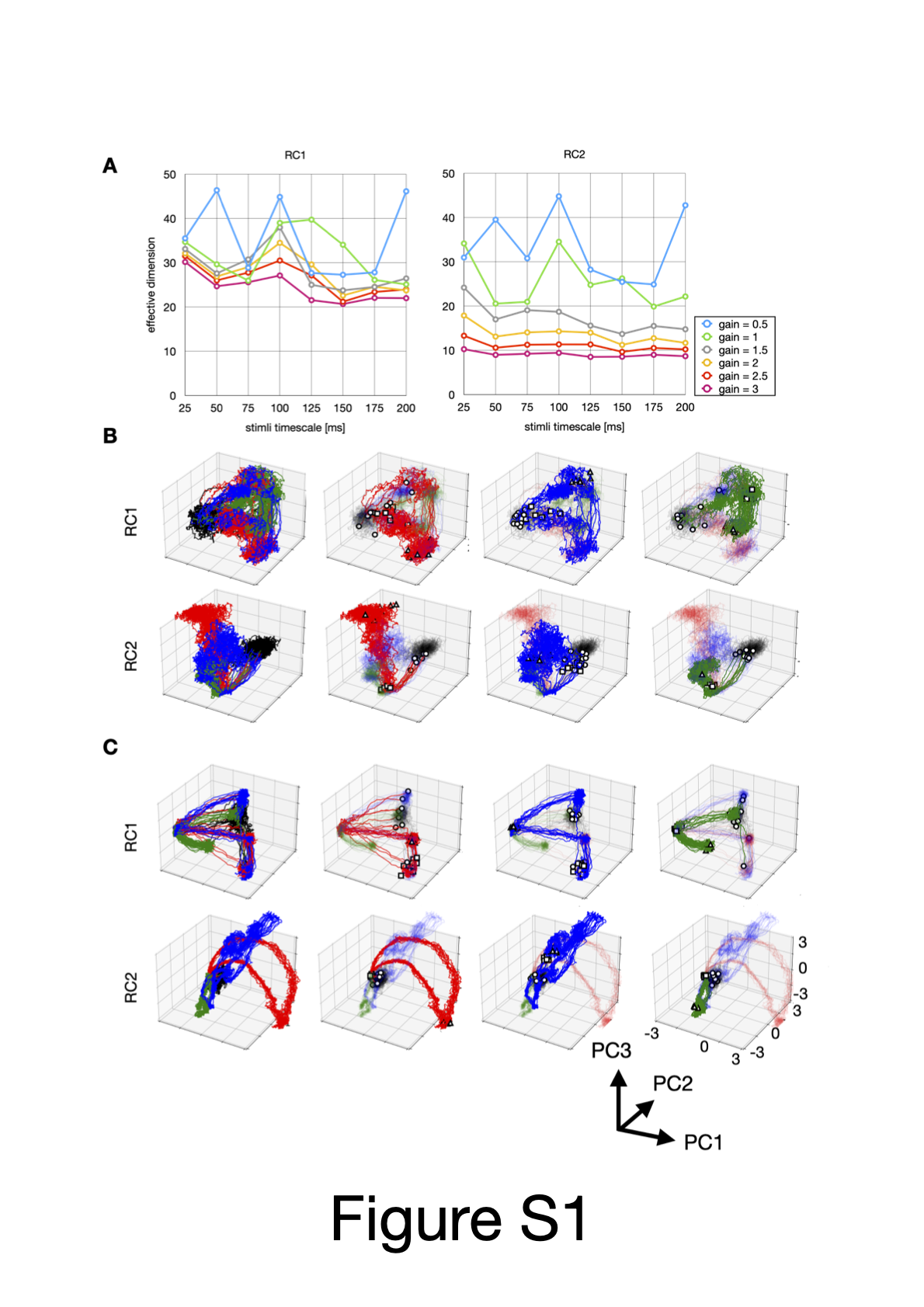


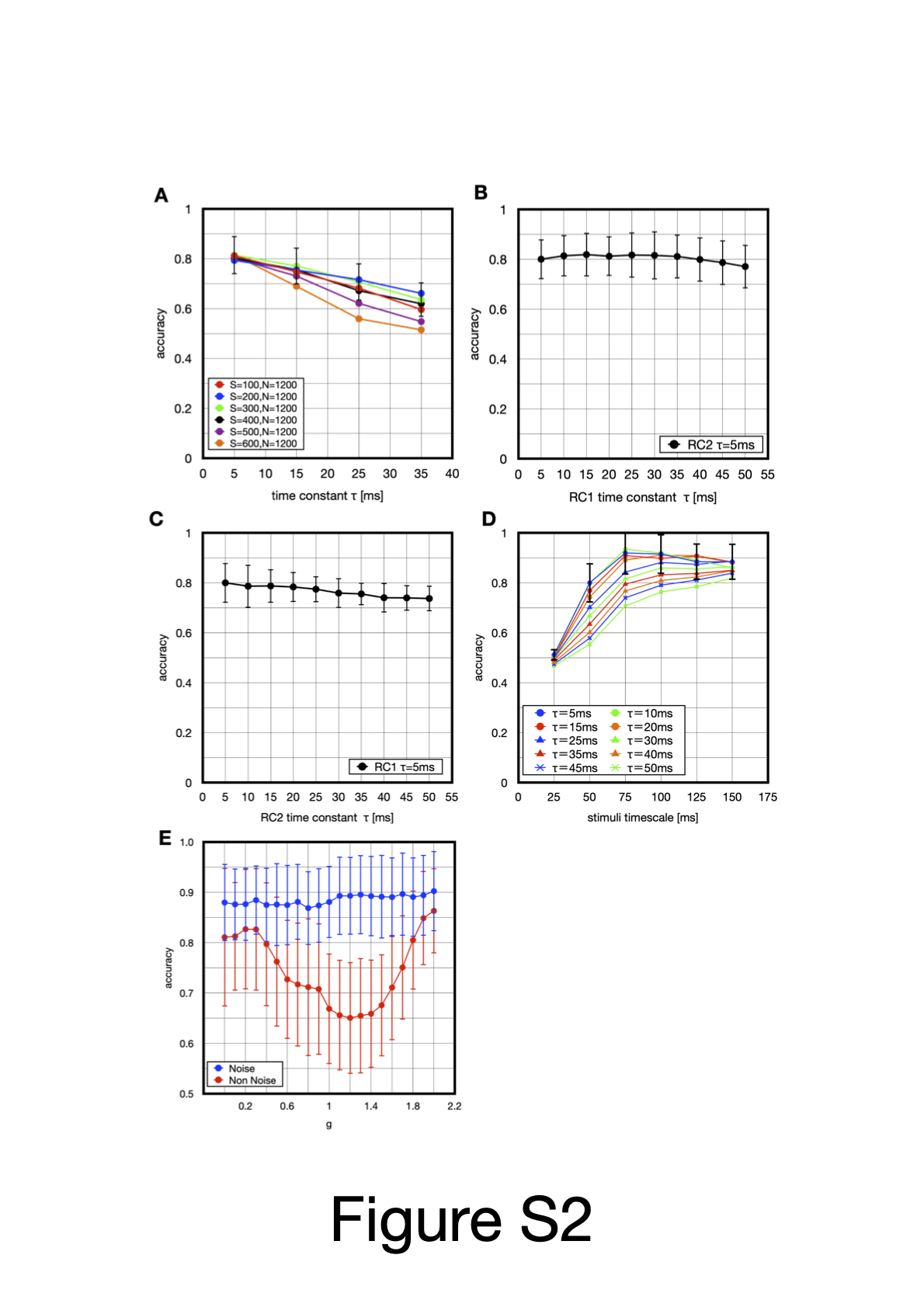


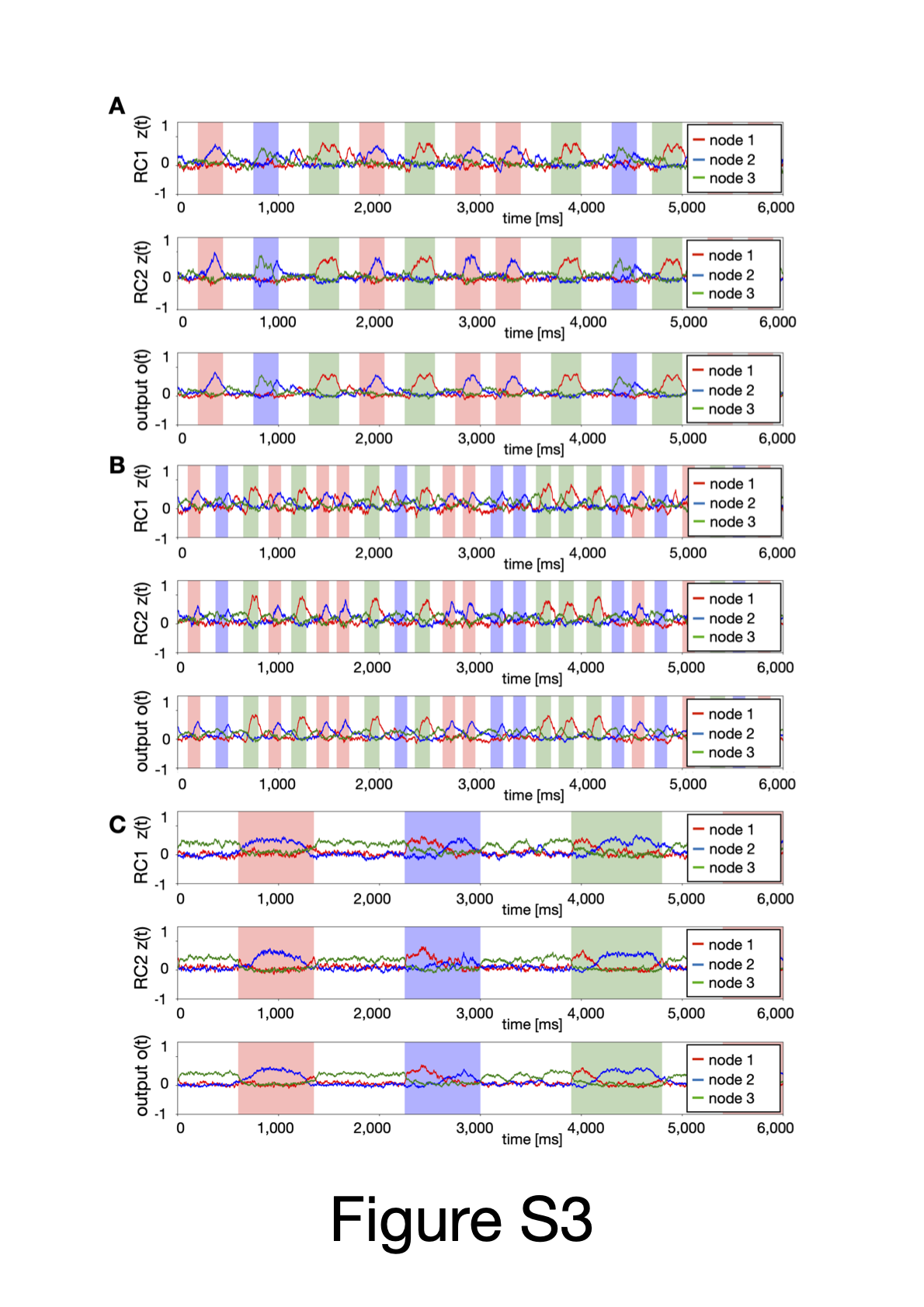


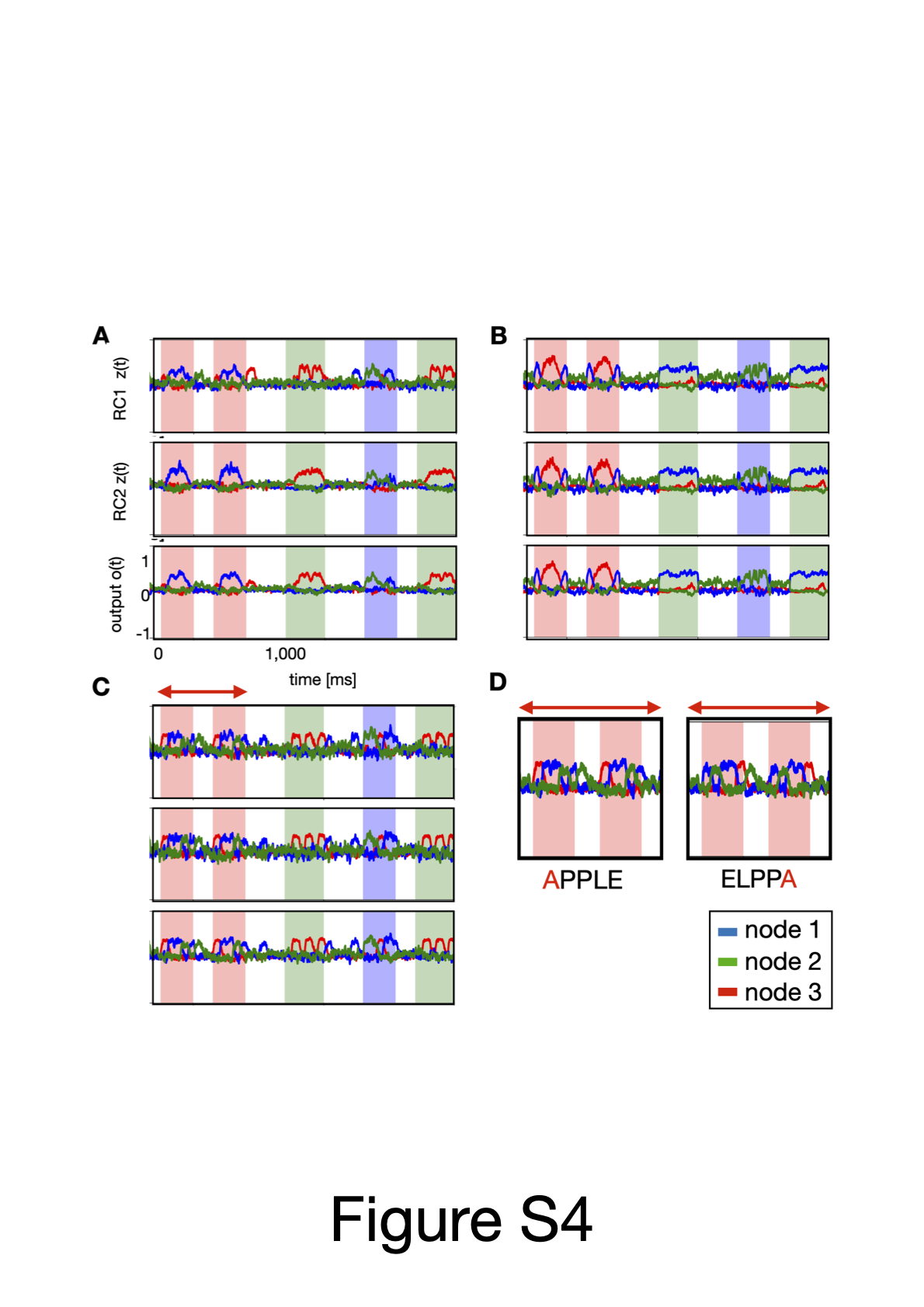


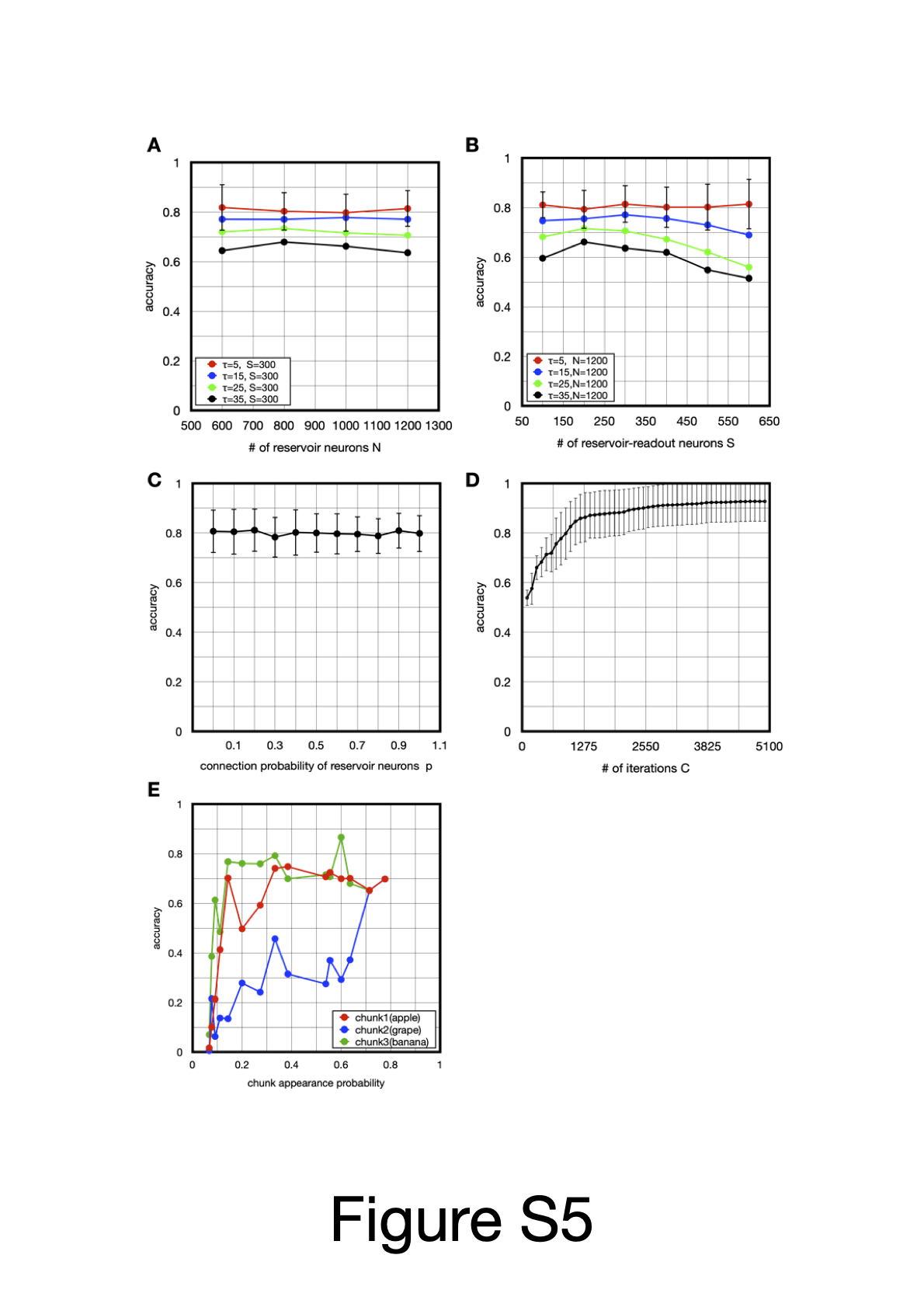

Supplement: Supplementary file 1 — Supplementary Information. [file 41598_2023_27385_MOESM1_ESM.docx]
